# Resting-state oscillations reveal disturbed excitation–inhibition ratio in Alzheimer’s disease patients

**DOI:** 10.1038/s41598-023-33973-8

**Published:** 2023-05-07

**Authors:** Anne M. van Nifterick, Danique Mulder, Denise J. Duineveld, Marina Diachenko, Philip Scheltens, Cornelis J. Stam, Ronald E. van Kesteren, Klaus Linkenkaer-Hansen, Arjan Hillebrand, Alida A. Gouw

**Affiliations:** 1 Alzheimer Center Amsterdam, Neurology, Vrije Universiteit Amsterdam, Amsterdam UMC location VUmc, Amsterdam, The Netherlands; 2 Clinical Neurophysiology and MEG Center, Neurology, Vrije Universiteit Amsterdam, Amsterdam UMC location VUmc, Amsterdam, The Netherlands; 3 Department of Integrative Neurophysiology, Center for Neurogenomics and Cognitive Research, Vrije Universiteit Amsterdam, 1081 HV Amsterdam, The Netherlands; 4 Amsterdam Neuroscience, Neurodegeneration, Amsterdam, The Netherlands; 5 Amsterdam Neuroscience, Systems and Network Neurosciences, Amsterdam, The Netherlands; 6 Department of Molecular and Cellular Neurobiology, Center for Neurogenomics and Cognitive Research, Vrije Universiteit Amsterdam, 1081 HV Amsterdam, The Netherlands

**Keywords:** Diagnostic markers, Inhibition-excitation balance, Alzheimer's disease, Neurophysiology

## Abstract

An early disruption of neuronal excitation–inhibition (E–I) balance in preclinical animal models of Alzheimer’s disease (AD) has been frequently reported, but is difficult to measure directly and non-invasively in humans. Here, we examined known and novel neurophysiological measures sensitive to E–I in patients across the AD continuum. Resting-state magnetoencephalography (MEG) data of 86 amyloid-biomarker-confirmed subjects across the AD continuum (17 patients diagnosed with subjective cognitive decline, 18 with mild cognitive impairment (MCI) and 51 with dementia due to probable AD (AD dementia)), 46 healthy elderly and 20 young control subjects were reconstructed to source-space. E–I balance was investigated by detrended fluctuation analysis (*DFA*), a functional E/I (*fE/I*) algorithm, and the aperiodic exponent of the power spectrum. We found a disrupted E–I ratio in AD dementia patients specifically, by a lower *DFA*, and a shift towards higher excitation, by a higher *fE/I* and a lower aperiodic exponent. Healthy subjects showed lower *fE/I* ratios *(*< 1.0) than reported in previous literature, not explained by age or choice of an arbitrary threshold parameter, which warrants caution in interpretation of *fE/I* results. Correlation analyses showed that a lower *DFA* (E–I imbalance) and a lower aperiodic exponent (more excitation) was associated with a worse cognitive score in AD dementia patients. In contrast, a higher *DFA* in the hippocampi of MCI patients was associated with a worse cognitive score. This MEG-study showed E–I imbalance, likely due to increased excitation, in AD dementia, but not in early stage AD patients. To accurately determine the direction of shift in E–I balance, validations of the currently used markers and additional in vivo markers of E–I are required.

## Introduction

While previous studies have frequently reported cortical and hippocampal neuronal hyperactivity in preclinical animal models of Alzheimer’s disease (AD)^1–3^, hypoactive and silent neurons have been found in late stage models of Alzheimer’s disease (AD) with profound amyloid plaque formations^2,4^. In addition, tau pathology has been predominantly related to neuronal silencing and inhibition of the neuronal network^5,6^. These findings show a disruption in the balance between excitation and inhibition (E–I) as the disease progresses, that can lead to brain network dysfunction and cognitive impairment^7,8^. If, when and how such a shift occurs in human AD patients has been difficult to investigate, because obtaining direct evidence of E–I ratio changes in living human subjects is challenging. Noninvasive electromagnetic recordings directly capture the activity of larger populations of pyramidal neurons and inhibitory interneurons and, thus, offers a way to study shifts in E–I ratio.

Electroencephalography (EEG) and magnetoencephalography (MEG) studies have already showed signs of neuronal hyperexcitability in AD patients, by a higher risk of seizures^9–12^ and subclinical epileptiform discharges^13,14^. AD patients with epileptiform activity also presented a faster decline in cognitive function^13,14^, highlighting the relevance to identify such abnormalities at an early stage. Although (subclinical) epileptiform abnormalities are the most overt sign of neuronal hyperexcitability, which in turn is a result of E–I ratio changes, they occur infrequently, locally and predominantly during sleep^13–15^, and, therefore, challenges a correct detection. In addition, the vast number of automatic spike detection algorithms developed for neurophysiological data are not widely used in the clinic, yet, because their accuracy needs improvement^16–18^. Novel, quantitative surrogate markers of E–I balance are, thus, required.

Short resting-state electromagnetic recordings are more widely available and quantitative measures have been associated with neuronal activity in AD patients^19–21^, including spectral power^22^ and functional connectivity^19,23^, among others^24–26^. Computational modeling studies have provided additional evidence that macroscale recordings provide information about E–I balance in AD^27–29^. Such metrics may thus provide a translational link between the E–I balance at a single neuron or local circuit scale, as frequently studied in preclinical AD animal models, and the macroscale cortical network dynamics, as measured in human AD patients^30^. Two metrics are of special interest because they do not only show an imbalance, but also inform us about the direction of change in E–I. The aperiodic, 1/f-like, exponent of the power spectrum^31,32^, in the gamma frequency range in particular, can track shifts in E–I ratio at many spatial scales^33^. Higher excitatory activity was related to a lower aperiodic exponent, reflecting a flattened slope^33^. In addition, a novel algorithm has been proposed that computes a functional E–I ratio (*fE/I*) from the spectro-temporal properties of neuronal network oscillations^34^. The *fE/I* was ~ 1 in the EEG of a healthy adult population and abnormal after pharmacological intervention and in a clinical population. The *fE/I* thus detects shifts in E_I ratios, such that more excited or inhibited networks show an *fE/I* > 1 or < 1, respectively^34^.

In this study, we determined both the aperiodic (1/f-like) exponent of the power spectrum and the *fE/I* of resting-state MEG data to investigate whether patients across different stages of AD have an opposite change in E–I ratio, e.g. an early increase and late decrease, as suggested by preclinical data. We also performed detrended fluctuation analyses (*DFA*) to quantify long-range temporal correlations, because it is sensitive to changes in E–I ratio^35^, previously reported to be altered in early-stage AD^36^, and because it is a component of the *fE/I* analyses. It is hypothesized that the healthy brain operates near a critical point, characterized by maximal *DFA,* which is essential for optimal information processing^37^. A diseased brain, that operates further from the critical point, due to an excess of inhibition or excitation, is expected to show sub- or supercritical dynamics, respectively, which is shown as lower *DFA* values^37–39^. Unlike the aperiodic exponent and the *fE/I* measure, *DFA* indicates whether the network is out of balance, but, thus, does not inform about the direction in which E–I has changed. We examined three indicators of E–I balance in parallel, so that the indices can reinforce each other and provide a validation of the novel markers. We also studied MEG data of healthy young (HY) and elderly (HE) subjects as reference groups, for whom we expected to find a balanced network. Detecting AD patients with excessively activated or inactivated network has important implications as potential predictor of the rate of cognitive decline and the selection of patients whom are eligible for participation in future clinical trials that restore E–I balance.

## Results

### Subject characteristics

A summary of the participant characteristics per group is given in Table 1. Groups were similar in sex distribution (*X*^*2*^(4) = 4.69, *p* = 0.321) but differed in mean age (*F*(4,146) = 115.92, *p* < 0.001), education level (*F*(4,146) = 6.63, *p* < 0.001) and mini-mental state examination (MMSE) score (*F*(3,126) = 36.24, *p* < 0.001). Pairwise comparisons showed that the HY group was (by construction) significantly younger compared to all other groups (*p* < 0.001) and HE was significantly younger than the group with mild cognitive impairment (MCI) (*p* = 0.001) and dementia due to AD (AD) (*p* < 0.001). HY had a significantly higher education level compared to HE (*p* = 0.009) and AD (*p* < 0.001). AD had, as expected, a significantly lower MMSE score than HE, SCD, MCI and AD (all *p* < 0.001).

**Table 1:** Subject characteristics.

	HY	HE	SCD	MCI	AD	*p*-value
N	20	45	17	18	51	
Sex (m/f)	10/10	28/18	9/8	11/0	23/28	0.321
Age (years)	25 ± 3.1	57 ± 8.4	60 ± 8.0	65 ± 5.9	64 ± 7.6	< 0.001
Education	6.5 ± 0.6	5.4 ± 1.4	5.5 ± 1.0	5.4 ± 1.1	5.1 ± 1.1	< 0.001
MMSE	n.a	28 ± 1.8	27 ± 3.3*	26 ± 2.1	20 ± 5.3^#^	< 0.001

Values are presented as mean ± standard deviation (SD). Statistical analyses were performed using the chi square test (sex) and one-way ANOVA (others).

*HY* healthy young controls, *HE* healthy elderly controls, *SCD* subjective cognitive decline, *MCI* mild cognitive impairment, *AD* dementia due to Alzheimer’s disease, *m/f* male/female, *y* years, *Education* Verhage score (range 1–7), *MMSE* mini-mental state examination (range 0–30), *n.a.* not applicable.

*Available for *n* = 16, ^#^available for *n* = 50.

### AD patients show oscillatory slowing

**Figure 1:**
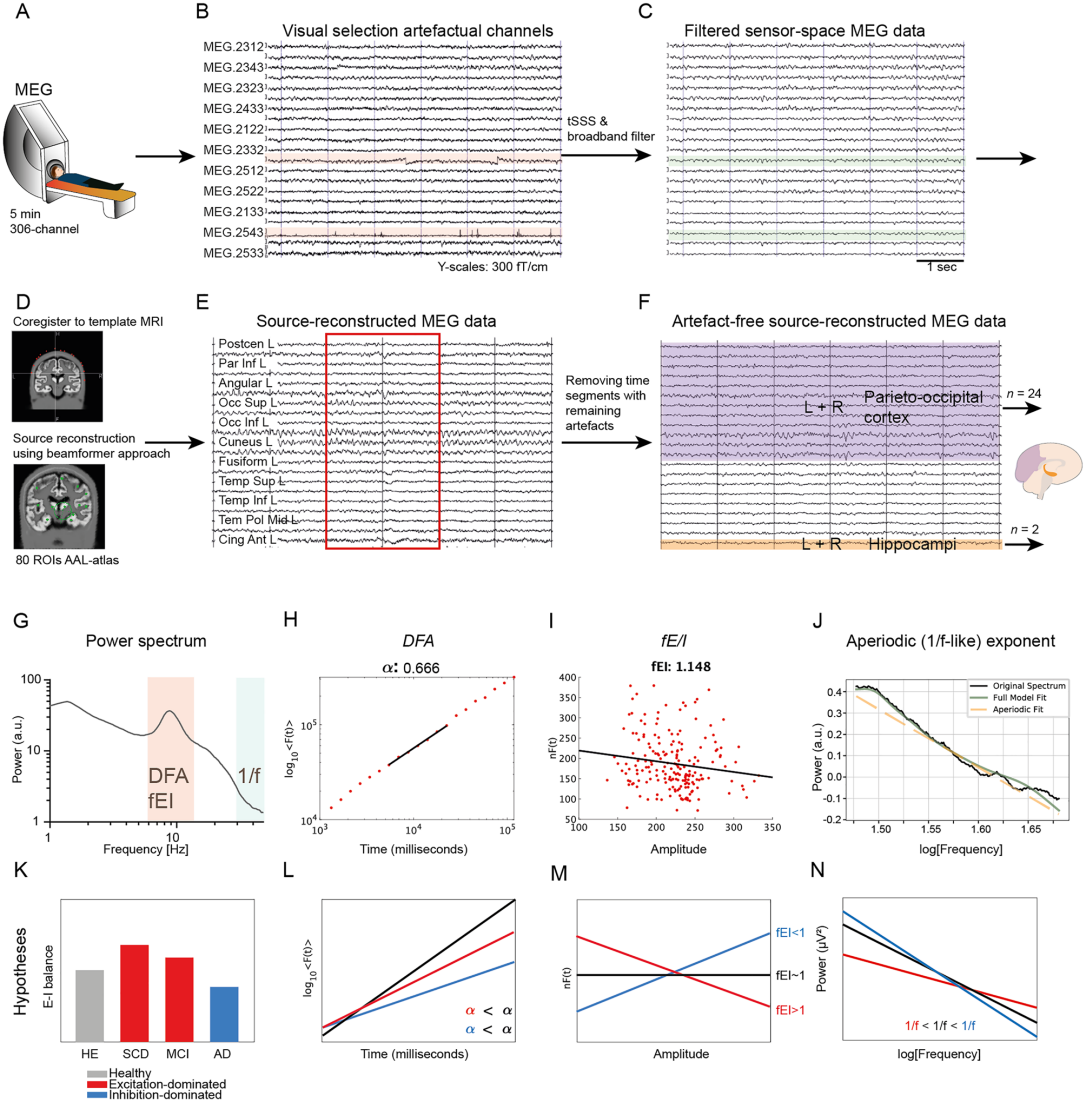
Schematic of MEG data preprocessing and analyses. (**A**–**D**) Standardized resting-state MEG data preprocessing procedures. (**E**) Visual selection and removal of time segments with remaining artefacts (red box), drowsiness or eyes-open. (**F**) Subsequent analyses of clean source-space MEG time series were restricted to the parieto-occipital cortex (Supplementary Table S1) and the left and right hippocampi. (**G**) Power spectra (1–48 Hz) were obtained by means of a Fast Fourier Transform. (**H**) Long-range temporal correlations in the extended alpha band (6–13 Hz) were estimated using the *DFA* algorithm. To obtain the *DFA* exponent, the algorithm fits a slope, *α,* to the mean fluctuations of the signal profile (*y*-axis) across log-spaced time windows (*x*-axis). (**I**) The *fE/I* algorithm correlates the average amplitude alpha band oscillations (*x*-axis) to the amplitude-normalized fluctuation functions (*nF*(t), *y*-axis) calculated in 5-s windows. (**J**) The aperiodic exponent of the power spectrum in the gamma frequencies (30–48 Hz) was obtained using the *FOOOF* algorithm. (**K**) Hypotheses for E–I (im)balance in AD patients. (**L**–**N**) Hypothesized *DFA* exponents, *fE/I* values and aperiodic exponents in healthy (*black*), excitation-dominated (*red*) and inhibition-dominated (*blue*) states. *MEG* Magnetoencephalography, *MRI* magnetic resonance imaging, *tSSS* temporal extension of signal space separation, *ROIs* regions of interest, *AAL-atlas* automated anatomical labeling atlas, *L* + *R* left and right hemisphere, *DFA* detrended fluctuation analyses, *fE/I* functional excitation/inhibition, *HE* healthy elderly controls, *SCD* subjective cognitive decline, *MCI* mild cognitive impairment, *AD* dementia due to Alzheimer’s disease.

**Figure 2:**
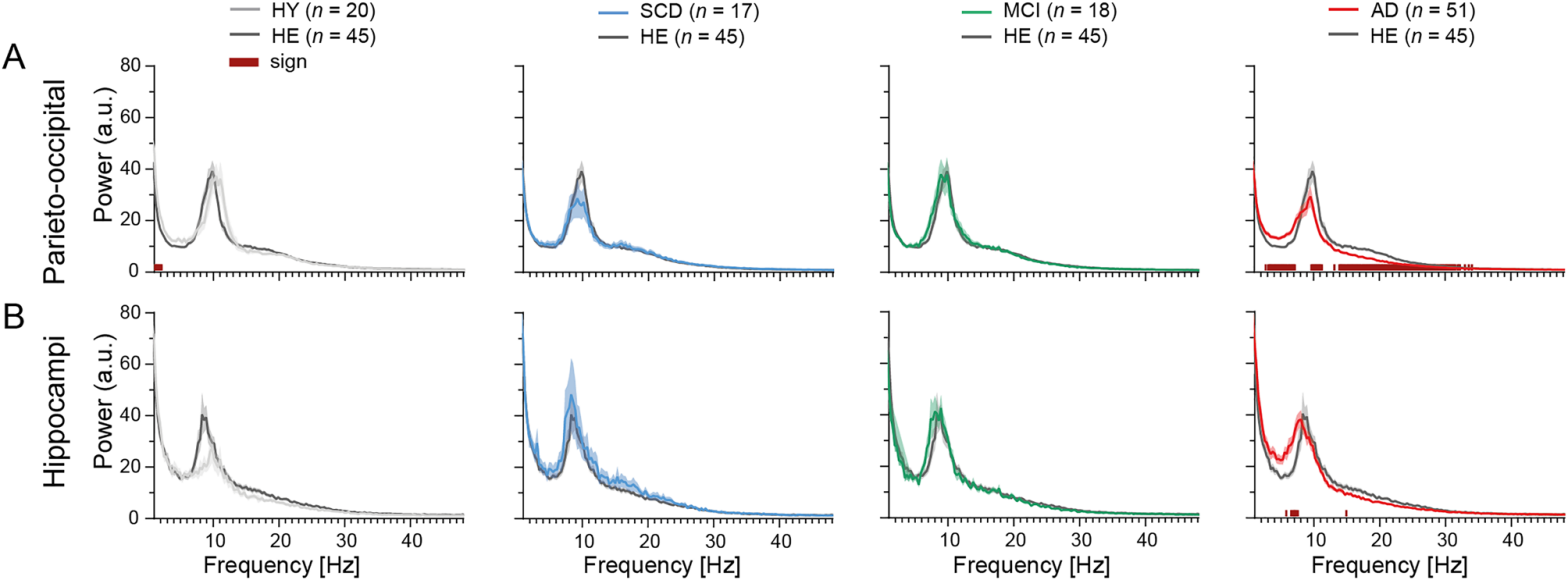
Oscillatory slowing in AD patients, but not in MCI or SCD patients. The mean power spectrum (1–48 Hz) in the parieto-occipital cortex (**A**) and in the hippocampi (**B**). The power per frequency bin was compared between each group (HY, SCD, MCI, AD) and HE, respectively. Shaded area around each curve shows standard error of the mean. Statistical analyses were performed using multiple Mann–Whitney U-tests and *FDR* corrected. Dark red bars on the *x*-axis present significant differences. *HY* healthy young controls, *HE* healthy elderly controls, *SCD* subjective cognitive decline, *MCI* mild cognitive impairment, *AD* dementia due to Alzheimer’s disease.

An overview of the MEG data preprocessing and analyses steps, as well as the hypothesized outcomes are provided in Fig. 1. We first evaluated spectral power across a range of frequencies (1–48 Hz). The mean power of each frequency bin for each group (HY, SCD, MCI, AD) was compared to HE (*FDR* corrected) (Fig. 2). In the parieto-occipital cortex, AD patients showed significantly higher power in the lower frequencies (~ 3.1–7.2 Hz) and significantly lower power at higher frequencies (~ 9.7–11.3 Hz and ~ 14.0–32.2 Hz). Similar power differences between HE and AD, but across a smaller frequency range, were found in the hippocampi. No differences were found between SCD or MCI compared to HE. HY had significantly higher power in the low delta frequencies (~ 1.1–2.2 Hz) in (only) the parieto-occipital cortex compared to HE.

### AD patients have decreased long-range temporal correlations

**Figure 3:**
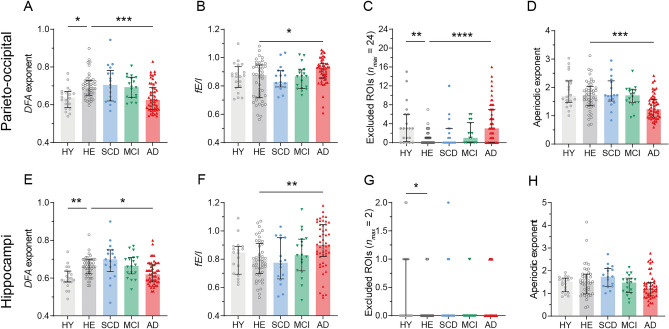
Abnormal E–I balance in AD patients, but not in MCI or SCD patients. (**A**,**E**) Lower *DFA* exponents in the extended alpha band (6–13 Hz) in parieto-occipital cortex and hippocampi suggest E–I imbalance in AD patients and HY compared to HE. (**B**,**F**) *fE/I* values of ~ 1.0 indicate E–I balance, but many HE and HY subjects showed *fE/I* < 1.0, which suggest inhibition-dominated activity. Median *fE/I* was relatively higher in AD patients compared to HE, suggesting less inhibition, but more balanced networks. (**C**,**G**) The number of ROIs for which no *fE/I* value was calculated (because *DFA* < 0.55) was higher in AD and HY than HE. (**D**,**H**) The aperiodic exponent of the gamma frequency power spectra was lower in AD, suggesting relatively more excitation in the parieto-occipital cortex compared to HE. Each symbol shows the average of all ROIs within the parieto-occipital cortex or the hippocampi of a single subject. Bar graphs show the median and interquartile range per group. Outcome measures for each group (HY, SCD, MCI, AD) were compared to HE using the Kruskal–Wallis test. *DFA* detrended fluctuation analyses, *fE/I* functional excitation inhibition, *ROIs* region of interest, *HY* healthy young controls, *HE* healthy elderly controls, *SCD* subjective cognitive decline, *MCI* mild cognitive impairment, *AD* dementia due to Alzheimer’s disease; *p < 0.05; **p < 0.01, ***p < 0.001.

We next investigated whether long-range temporal (auto)correlations in the MEG signal were different in patients across the AD continuum compared to healthy controls (Fig. 1H,L). To do so, the average alpha-band (6–13 Hz) *DFA* in the parieto-occipital cortex and hippocampi for each subject was calculated (Fig. 3A,E). No outliers were detected in any of the groups. *DFA* exponents of the parieto-occipital cortex were significantly different between groups (*H*(4) = 24.51, *p* < 0.001). Pairwise analyses revealed a lower *DFA* exponent in AD patients (mean rank 57.57) compared to HE (mean rank 93.44) (*p* < 0.001), indicating weaker autocorrelations in the parieto-occipital signal in AD. Interestingly, HY (mean rank 56.75) also had significantly lower *DFA* exponents than HE (*p* = 0.007). *DFA* exponents of the hippocampi also differed between groups (*H*(4) = 26.19, *p* < 0.001), and similar group differences were found in *DFA* exponents of the hippocampus: lower *DFA* in AD patients (mean rank 62.19) and HY (mean rank 46.83) than in HE (mean rank 88.93) (*p* = 0.011 and *p* = 0.002, respectively).

We also evaluated parieto-occipital *DFA* exponents across a broader range of frequencies (range 1–44 Hz with steps of 1 Hz) (Supplementary Fig. S2A). *DFA* exponents were generally highest in the (extended) alpha band in all groups, although a peak in the beta frequency band (13 – ~ 25 Hz) was also observed. Except for the beta band, group differences were generally smaller in size and of similar direction as found for the alpha band (lower *DFA* in AD and HY compared to HE) (Supplementary Fig. S2B). Of note, average *DFA* exponents in gamma frequencies (> 30 Hz) were generally < 0.55, thus, showing no long-range temporal correlations.

### AD patients have relatively higher excitation–inhibition balance

To test whether patients across the AD continuum have an early increase and late decrease in E–I balance, we assessed the *fE/I* (Fig. 3B,F). Although no outliers were detected, for *n* = 2 HY subjects and *n* = 1 SCD patient no hippocampal *fE/I* values were calculated, because both ROIs had *DFA* exponents < 0.55 (see methods). In contrast to an average whole-brain *fE/I* value of ~ 1 in healthy adults as previously observed in EEG data^34^, both the HE and HY subjects showed a median *fE/I* < 1.0 in both the parieto-occipital cortex (median(Q1–Q3): HY, 0.86 (0.79–0.94); HE, 0.88 (0.72–0.95)) and hippocampi (median(Q1–Q3): HY (*n* = 18), 0.85 (0.69–0.89); HE, 0.79 (0.69–0.91)). An *fE/I* < 1.0 has been previously related to pathological network imbalance, and an inhibition-dominated regime specifically^34,40^. *fE/I* values were not calculated for ROIs with a *DFA* < 0.55^34^. The number of ROIs with *DFA* < 0.55 in the parieto-occipital cortex differed significantly between groups (*H*(4) 26.23,* p* < 0.001) (Fig. 3C,G). Pairwise analyses showed that this number was, compared to HE, higher in AD patients (mean rank 94.98) than in HE (mean rank 54.31) and HY (mean rank 90.93), consistent with the *DFA* findings reported above. The number of ROIs with *DFA* < 0.55 in the hippocampi was not significantly different between groups (*H*(4) = 7.830, *p* = 0.098), but pairwise tests revealed a higher number of excluded ROIs in HY (mean rank 90.20) compared to HE (mean rank 69.58).

Groups did not significantly differ in *fE/I* in the parieto-occipital cortex (*H*(4) = 9.365,* p* = 0.053), but pairwise analyses showed that AD patients had a significantly higher *fE/I* (mean rank 91.16) compared to HE (mean rank 67.62) in the parieto-occipital cortex (*p* = 0.034). A significant group difference in *fE/I* was found in the hippocampus (*H*(4) = 14.59, *p* = 0.006) and pairwise analyses revealed a higher *fE/I* in the hippocampi (mean rank 68.56) in AD patients compared to HE (62.20) (*p* = 0.003) (Fig. 3B,F). While their *fE/I* values were closer to 1, suggesting more balanced E–I than HE, the relatively increased *fE/I* values also suggest that AD patients have more excitation than HE. Although the excluded number of ROIs was higher when using a more conservative *DFA* threshold of 0.6, group differences were similar (Supplementary Fig. S1).

We also examined *fE/I* across a broader range of frequencies (range 1–44 Hz with steps of 1 Hz) (Supplementary Fig. S2C). For all groups, *fE/I* values were closer to 1.0 for delta-theta (~ 1–6 Hz) and gamma frequencies (~ 30–40 Hz). However, the number of ROIs for which *fE/I* could not be obtained was high (> 50%) due to *DFA* < 0.55 in the gamma frequencies especially (Fig. 1E). Group differences between AD patients and HE were largest and of similar direction in alpha and beta frequencies (not statistically tested, Supplementary Fig. S2D). Differences between MCI and HE seemed largest in the high alpha and beta frequencies (~ 12–10 Hz), a frequency range not captured in the extended alpha band (6–13 Hz).

### AD patients have a lower aperiodic gamma exponent

An alternative method to determine a difference in E–I balance is the aperiodic exponent of the power spectrum in the gamma frequency range (30–48 Hz) (Fig. 3D,H). One outlier was detected in the parieto-occipital aperiodic exponent in the HE group and removed before analysis. In addition, aperiodic exponents of the parieto-occipital cortex in *n* = 2 AD patients and aperiodic exponents of the hippocampi in *n* = 2 HE, *n* = 1 SCD and *n* = 6 AD subjects were excluded due to a bad fit (*R*^*2*^ < 0.8). The aperiodic exponent was significantly different across groups in the parieto-occipital cortex (*H*(4) = 26.16, *p* < 0.001), and pairwise tests showed that the exponent was lower in AD patients (mean rank 49.37) compared to HE (mean rank 83.75) (*p* < 0.001). The aperiodic gamma exponent was also different across groups in the hippocampus (*H*(4) = 9.863, *p* = 0.043). AD patients showed lower exponents (mean rank 58.91) than HE (mean rank 76.07), but this difference was not statistically significant (*p* = 0.202). A lower exponent in the gamma frequency range of the parieto-occipital cortex suggest a higher E–I ratio in AD patients.

### MEG based measures of E–I balance correlate with cognitive scores

**Figure 4:**
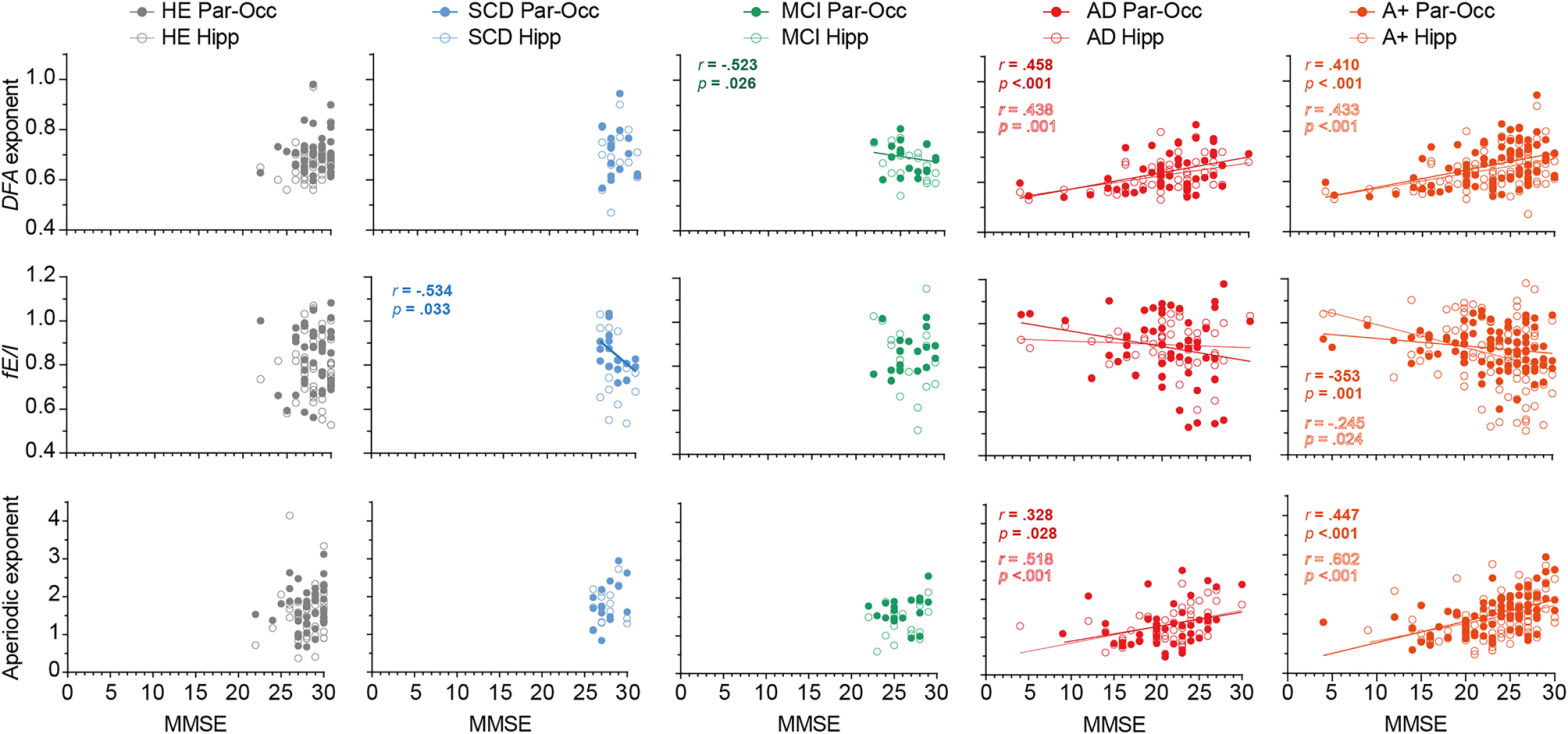
E–I measures correlate to cognitive score in amyloid-positive subjects. E–I measures (*DFA (first row)*, *fE/I (second row)* and aperiodic exponent (*third row*)) per group were plotted as function of global cognition as measured by the mini-mental state examination (MMSE) scores. If the Spearman’s rank correlation analyses showed a statistically significant correlation between the E–I measure and the MMSE score (Table 2), a linear regression line and the *r-* and *p*-values were presented. *MMSE* mini-mental state examination, *DFA exponents* detrended fluctuation analyses exponent, *fE/I* functional excitation/inhibition, *HE* healthy elderly controls, *SCD* subjective cognitive decline, *MCI* mild cognitive impairment, *AD* dementia due to Alzheimer’s disease, *Par-Occ* parieto-occipital cortex, *Hipp* hippocampi.

To explore the relationship between the E–I outcome measures and the clinical disease stage of patients across the AD continuum, we analyzed the correlation between each measure of E–I and global cognitive (MMSE) scores (Fig. 4, Table 2). A Spearman’s rho correlation analyses showed that within all subjects with proven amyloid pathology, all three measures of E–I correlated significantly with MMSE scores in the parieto-occipital cortex as well as the hippocampi. When stratifying for clinical diagnostic group, significant positive correlations were found between MMSE scores and *DFA* exponents and between MMSE scores and aperiodic gamma exponent in AD patients in the parieto-occipital cortex and hippocampi. In addition, MMSE scores negatively correlated to hippocampal *DFA* exponents in MCI and parieto-occipital *fE/I* values in SCD.

**Table 2:** Spearman correlations between E–I measures and MMSE scores.

		Parieto-occipital cortex			Hippocampi		
		*DFA*	*fE/I*	Aperiodic exponent	*DFA*	*fE/I*	Aperiodic exponent
HE	*n*	45	45	44	45	45	43
	*r*	0.037	0.058	0.174	0.201	− 0.109	0.167
	*p*-value	0.811	0.703	0.260	0.186	0.476	0.284
SCD	*n*	16	16	16	16	15	15
	*r*	− 0.114	− 0.534	0.379	0.126	− 0.325	0.103
	*p*-value	0.674	0.033	0.148	0.641	0.237	0.715
MCI	*n*	18	18	18	18	18	18
	*r*	− 0.173	0.338	0.295	− 0.523	− 0.371	0.353
	*p*-value	0.492	0.170	0.235	0.026	0.130	0.151
AD	*n*	50	50	48	50	50	45
	*r*	0.438	− 0.046	0.518	0.458	− 0.176	0.328
	*p*-value	0.001	0.753	< 0.001	< 0.001	0.220	0.028
A +	*n*	84	84	82	84	83	78
	*r*	0.433	− 0.245	0.602	0.410	− 0.353	0.447
	*p*-value	< 0.001	0.024	< 0.001	< 0.001	0.001	< 0.001

*DFA* detrended fluctuation analyses, *fE/I* functional excitation/inhibition ratio, *HE* healthy elderly controls, *SCD* subjective cognitive decline, *MCI* mild cognitive impairment, *AD* dementia due to Alzheimer’s disease, *A* + all amyloid-biomarker-positive patients, *Par-Occ* parieto-occipital cortex, *Hipp* hippocampi.

## Discussion

Opposite to findings from preclinical research in AD animal models, this MEG study could not provide evidence for an abnormally high E–I ratio in early stage AD patients using three measures in parallel. Instead, all three measures consistently showed abnormal E–I ratio in AD dementia patients. Two of these E–I markers (*fE/*I and aperiodic exponent) that also indicate the direction of change, suggested relatively more excitation in AD patients compared to healthy elderly. Healthy elderly and young subjects however showed abnormally low *fE/I* values and AD dementia patients *fE/I* values more close to 1, which should indicate more balanced networks. These findings, among other considerations explained below, warrant caution in the interpretation of *fE/I* as valid E–I ratio indicator in resting-state source-reconstructed MEG data and highlight the need to provide further validation of the used measures as well as additional non-invasive metrics of E–I (im)balance.

Previous studies have shown that oscillatory slowing of MEG signals, a robust electromagnetic signature of AD^41,42^, starts already in amyloid-biomarker positive subjects diagnosed with MCI^43,44^ or, even earlier, in SCD patients^43,45^. In the current study, AD patients showed clear oscillatory slowing (i.e., reduced alpha/beta power and increased delta/theta power) in both the parieto-occipital cortex and the hippocampi, but no spectral abnormalities were detected in SCD or MCI. In addition, HY subjects showed significantly higher power in the delta frequencies of the parieto-occipital cortex, which is likely an age-related maturation effect^46–48^. A potential explanation for the lack of spectral power changes in the MCI and SCD patients is that we limited the study to the parieto-occipital cortex and hippocampi, while temporal ROIs and the whole-brain are known to show early signs of slowing^42,45,49^. In addition, we have a relatively small number of subjects in the SCD and MCI groups, which limits the probability to detect effects. Although spectral slowing, as observed in AD patients here, is intuitively associated with reduced E–I balance, a number of computational modeling studies showed a relation to increased excitatory neuronal activity instead^27,28,50^. This hypothesis requires verification in experimental studies, but, if true, the results fit with the observed increase in *fE/I* and reduction of aperiodic exponent, both suggesting higher levels of excitation in AD patients.

*DFA* results showed a lower temporal correlation in the amplitude fluctuations of the parieto-occipital cortex and the hippocampi in both AD patients and HY subjects compared to healthy elderly. In line with a lack of difference in spectral power, *DFA* values did not differ between SCD or MCI patients compared to healthy elderly. Lower *DFA* exponents have been reported before in AD patients and likely indicate that the underlying network is operating away from the critical point (where there is a balance between excitation and inhibition)^37^. More specifically, Montez and colleagues found a lower *DFA* exponent of the temporo-parietal alpha band oscillations in MEG^36^, and Stam and colleagues reported a disruption of the fluctuations in the level of synchronization of resting-state EEG in AD patients^51^. In another (preprinted) MEG study, a significantly lower DFA widespread across the cortex was found in larger groups of SCD and MCI patients (without amyloid-biomarker confirmation) compared to controls The relatively small sample size of SCD and MCI patients in the current study may explain these contradictory findings. Interestingly, the effects were found across a similar frequency range as in AD patients here (in alpha, but also beta frequencies) and suggest a gradual decrease of *DFA* values across disease stages. The remarkably low *DFA* values found in healthy young subjects in the current study probably indicates the importance of taking structural connectivity differences between groups into account. The white matter volume of the brain increases up to the fourth or fifth decade of life^52,53^ and is especially disrupted in neurodegenerative disorders such as AD^54^. Future studies should, ideally, incorporate differences in structural brain abnormalities across subjects when interpreting measures of E–I, especially in neurodegenerative disorders. Overall, the *DFA* results of the current study are in line with previous reports in AD patients and suggest late-stage E–I imbalance.

Despite a lack of between-group difference, hippocampal *DFA* exponents in MCI patients showed a significant negative correlation to global cognition. MCI patients with worse cognitive scores, thus, had higher hippocampal *DFA* exponents. In contrast, correlation analyses showed that lower *DFA* exponents were associated with worse cognitive scores in AD patients. The opposite correlations between *DFA* exponents and global cognition in AD and MCI patients could be an indication for the biphasic hypothesis^50,55^. In particular, higher *DFA* values have been correlated to a shorter distance from seizure onset zones in human epilepsy patients, in temporal lobe intra-cortical EEG as well as in MEG of the parietal cortex^56,57^. This suggests that *DFA* exponents may provide information about the direction of E–I ratio change, such that higher *DFA* exponents reflect higher E–I ratios. However, how this exactly translates to between-subject differences in *DFA *values remains unknown. In addition, no negative correlations between cognition and other E–I measures were found in MCI patients. Future studies should find support for a plausible sub-critical (somewhat inhibited state) in healthy subjects, and, then, elucidate whether (relatively) increased *DFA* values reflect excitation-dominated, rather than more balanced, networks instead.*fE/I* values were higher in AD patients in the parieto-occipital cortex and the hippocampi, which suggest more excitation-dominated networks compared to healthy elderly. The significant negative correlation between hippocampal *fE/I* and global cognitive scores in patients with AD underscores these findings: worse cognitive performance was related to higher E–I ratios in the hippocampi. However, group level *fE/I* of both groups was < 1, which indicate inhibition-dominated networks, in contrast to what was expected for healthy controls based on previous work^34^. Another (preprinted) MEG study of *fE/I* ratios showed similarly low *fE/I* values in group of healthy elderly controls (mean age 70.21 years). The MCI patients showed similarly increased* fE/I* values, across similar (alpha and beta) frequencies^58^, as the AD patients in the current study. Two potential explanations for the low *fE/I* values in the healthy elderly of the current study is their age and subjective cognitive complaints. However, *fE/I* analyses in HY subjects (age range 20–30 years) without cognitive complaints showed similarly low *fE/I* values. Alternatively, healthy brains can never function exactly at the critical point^59^, but more likely operates somewhat below that point, in an inhibited or quasi-critical state^60^, which may have resulted here in an *fE/I* < 1. This is however ongoing research and cannot explain the normal *fE/I* values (close to 1) in sensor-space EEG analyses in healthy subjects^34^. Taken together, the unexpectedly low *fE/I* values in healthy subjects, the relatively normal *fE/I* values in AD patients, the large variance of *fE/I* values within groups, but also the higher number of excluded regions for *fE/I* analyses in AD patients, encourages nuance in the interpretation of the *fE/I* results.

Although the interpretation of the *DFA* and *fE/I* results across a broader range of frequencies (range 1–44 Hz with steps of 1 Hz) is not straightforward (see methods section), the group differences were, by visual inspection, largely consistent with the main (extended alpha band) findings. In addition, the beta frequencies presented the largest mean group differences between MCI patients and healthy elderly (although the number of ROIs with a *DFA* < 0.55 was generally higher compared to that of the alpha frequencies) and may guide future studies. Importantly, *fE/I* values are more close to 1 across gamma frequencies for all groups, as was also found in a previous (preprinted) MEG study^58^, but the large number of excluded ROIs (> 50%), probably due to the low SNR, and the lack of between-group differences makes this frequency range far from ideal to reliably capture *fE/I* ratio changes.

As already briefly mentioned, an important methodological issue of *fE/I* analyses that needs to be considered is the variability in number of ROIs per brain region for which one can calculate *fE/I*. Especially ROIs with more severely disrupted E–I balance will have time series without strong long-range temporal correlations (e.g., *DFA* exponents below 0.6), yet these ROIs were excluded from *fE/I* analysis. To reduce this bias, we chose an *DFA* exponent threshold of 0.55, which significantly increased the number of ROIs in the final *fE/I* analysis, while the main findings remained similar. Despite this approach, the number of excluded ROIs per brain region was significantly higher in AD patients, suggesting a larger number of ROIs with (severe) E–I imbalance. The higher *fE/I* in AD patients predominantly reflect the ROIs that have not (yet) been severely affected and that, perhaps, are in a relatively more excited state compared to elderly controls. Although this an interesting thought, the issue of *fE/I* dependence on *DFA* exponents is fundamental and requires optimization and additional analyses for future use in source-reconstructed MEG data.

Compared to controls, AD patients showed lower aperiodic exponents for the gamma frequency spectrum in the parieto-occipital cortex, indicating increased excitation. No differences were observed in SCD and MCI patients. Although the AD patients also showed non-significantly lower aperiodic gamma frequency exponents in the hippocampi, correlation analyses in both parieto-occipital and hippocampal regions showed that a lower aperiodic exponent was associated with a worse cognitive score. In a combined EEG/MEG and computational modeling (preprinted) study, that used the FOOOF-determined aperiodic exponent of the 1–40 Hz power spectrum and the model to estimate actual E–I ratios, a bidirectional change of the aperiodic exponent of the EEG power spectrum was found in AD patients, with decreased slopes (and higher E–I ratios) frontally, and increased slopes (and decreased E–I ratios) in temporal regions^61^. In MEG of MCI patients (without amyloid-biomarker confirmation) they found a unidirectional increase in E–I ratio (by a (not-significantly) reduced slope), across multiple regions, instead. Future studies should provide additional guidelines on the (aperiodic) range of frequencies that are most sensitive to E–I ratio changes and ROIs that are earliest affected in the course of AD.

The effect of ageing on the aperiodic exponent has been studied across several frequency ranges (2–25 Hz, 2–40 Hz, 20–150 Hz), and older ages were generally linked to lower aperiodic exponents in both EEG and MEG^62–64^. Although a direct comparison to these studies is difficult considering the differences in studied frequency ranges, ROIs and experimental task condition, we did not replicate these findings in the 30–48 Hz range: the HY subjects did not differ in aperiodic exponents compared to the elderly controls. One other (preprinted) study did also not find an association between age and resting-state EEG-based aperiodic exponent across the 2–30 Hz frequency range, but their age range (50–80 y) was smaller than those in the other studies^65^. Interestingly, their findings support the hypothesis that the aperiodic component is positively correlated to cognitive performance in healthy adults, as was also shown before in a large group of younger adults (18–40 y)^66^. Here, we found no correlation between aperiodic gamma exponent and cognitive score in healthy elderly, but a positive correlation in AD patients specifically, suggesting that this association was not merely an ageing effect.

The findings of this study should be interpreted with the following strengths and limitations in mind. We studied elderly subjects and patients that were well-characterized and had amyloid-biomarker confirmation. These groups and the addition of young controls give a full indication of the behavior of each metric across different ages and disease stages. A thorough cleaning procedure was adapted to remove artefacts, and E–I analyses was restricted to the parieto-occipital cortex, with high SNR in the alpha band oscillations, to increase the reliability of the *DFA* and *fE/I* metrics specifically. Because we used source-reconstructed MEG, we were able to also analyze the hippocampi which has great relevance in AD. The gamma frequency range of the aperiodic exponent was analyzed because this range showed substantial sensitivity to E–I ratio on multiple scales^33^. However, *fE/I* estimates are biased due to the dependence on *DFA* exponents. In addition, the aperiodic exponent of the gamma band may be influenced by environmental noise, even after the rigorous data cleaning. Furthermore, the absolute value of the aperiodic exponent can only be interpreted when compared to a reference group, which would complicate future clinical implementation. Groups significantly differed in age and education, but we have not replicated analyses while correcting for these nuance variables. A lack of significant results in the SCD and MCI group may have been a result of the low number of subjects and thus a lack of statistical power. The computation of each E–I outcome measure comes with several relatively arbitrary choices (e.g., think of fitting ranges in *DFA* exponents and aperiodic exponents) that likely influence results. Although others made a start in providing recommendations on how to more robustly estimate *DFA*^67^ and fit aperiodic exponents^68^, there is a need for additional guidelines to obtain consistent outcomes across different measures, modalities and scales (e.g., source- and sensor-space, or small- and large-scale networks). Finally, we did not identify ictal or interictal epileptiform activity as gold standard for increased E–I ratio in our population^21,69^.

## Methods:

### Participants

This retrospective study included a total of 86 patients across the AD continuum from the Amsterdam Dementia Cohort (ADC) (Van der Flier and Scheltens, 2018) of the Alzheimer Center Amsterdam at Amsterdam UMC, location VUmc, who underwent a magnetoencephalography (MEG) recording and had a known positive amyloid biomarker status through CSF and/or amyloid-PET imaging. All patients underwent extensive diagnostic screening, including neurological and neuropsychological examinations, magnetic resonance imaging (MRI), MEG, standard laboratory tests and a lumbar puncture (or amyloid Positron Emission Tomography (PET) scan). A multidisciplinary team established a clinical diagnosis according to the 2011 National Institute of Aging Alzheimer’s Association (NIA-AA criteria). Exclusion criteria were a medical history of significant neurological (i.e., other than dementia) or psychiatric disorders, proven early onset autosomal dominant AD, and use of acetylcholine-esterase inhibitors, antipsychotics, anti-epileptics, lithium or neuropathic pain medication at the time of MEG-measurement. This study involved 17 patients with subjective cognitive decline (SCD), 18 subjects with mild cognitive impairment (MCI) and 51 subjects with dementia due to probable Alzheimer’s disease (AD). Subjects included in the ADC with self-reported cognitive complaints but without objectively confirmed cognitive dysfunction and with negative amyloid biomarkers were considered healthy elderly controls (HE, *n* = 45)*.*

Although prior EEG data analyses in a group of 176 healthy subjects in the age range 19–56 years (mean 24.4 years, SD 7 years) validated the *fE/I* measure by reporting an whole-brain average *fE/I* of ± 0.99^34,40^, *fE/I* analyses have not been applied to MEG data or elderly subjects. Therefore, we also included a group of healthy young subjects (HY, *n* = 20) without (subjective) cognitive complaints of the MANTA study cohort of the Alzheimer Center Amsterdam (2018.070). These subjects underwent similar MEG and MRI as the elderly, and additional information about demographics and medication use was available. The local Medical Ethics Committee of the Amsterdam UMC location VUmc has approved a general protocol for biobanking and use of the clinical data for research purposes (2016.061; 2017.315). This study has been performed in accordance with the Declaration of Helsinki and relevant guidelines and regulations. All subjects gave written informed consent for use of their data for research purposes.

### MEG recordings

At least 5 min of eyes-closed resting-state MEG recordings were obtained in a magnetically shielded room using a 306-channel whole-head Vectorview MEG system (Elekta Neuromag Oy, Helsinki, Finland) at a sampling rate of 1250 Hz as part of the diagnostic setting^70^ or MANTA research protocol. Participants, who were in supine position, were instructed to relax but stay awake and reduce movements during the recording. An experienced technician continuously monitored the recording as well as the electro-oculogram to alert the patient by signs of drowsiness. When signs of drowsiness appeared, the subject was alerted through an acoustic signal or was instructed to shortly open the eyes. Electrocardiogram was also recorded. A 3D-digitizer (Fastrak, Polhemus, Colchester, VT, USA) digitized the position of five head localization coils, which were used to continuously determine the head position relative to the MEG sensors, as well as the scalp outline by approximately 500 points. The scalp surface was used for co-registration with a structural (MRI) template that produced the best fit^70^.

### MEG source-reconstruction

The temporal extension of Signal Space Separation (tSSS) implemented in the MaxFilter software (Elektra Neuromag. Oy, version 2.2.10) was applied to the raw sensor-space data to remove environmental artifacts. Before estimation of the SSS coefficients, bad channels (line noise, jump-artefacts, or flat signals) were visually identified and discarded (Fig. 1B). Source reconstruction was performed after applying a band-pass filter (0.5–100 Hz) to the tSSS-filtered data using an atlas-based centroid beamforming approach^71,72^ (Fig. 1C,D). The MEG signals were projected to 78 cortical and 2 hippocampal regions of interest (ROIs) according to the automated anatomical labeling (AAL) atlas^73^ (Supplementary Table S1). We determined the sphere that best fitted the scalp surface obtained from the co-registered MRI using surface matching (based on the Iterated Closest Point^74^ technique in combination with in-house developed software) and this sphere was used as a volume conductor model. The volume conductor model, an equivalent current dipole with optimum orientation^75^, and the MEG data covariance matrix (on average 300 s, range 227—343 s) were used to compute the broad-band (0.5–100 Hz) beamformer weights using a scalar beamformer as implemented in Elekta beamformer software (version 2.1.28). Singular value truncation was used when inverting the data covariance matrix to deal with the rank deficiency of the data after SSS, using a truncation limit of 1e^-6^ times the largest singular value. By projecting sensor-level MEG data through the normalized beamformer weights^76^, time series of neuronal activity were obtained for each ROI’s centroid. The source-reconstructed time series were converted to ASCII format for further analyses.

### MEG data analyses

After a visual inspection of the source-reconstructed data, time segments with remaining artefacts (not discarded by tSSS), such as eye blinks or muscle movements, eyes open or drowsy segments, were removed and the adjacent time series were concatenated (Fig. 1E,F). The final length of the time series differed per subject (137–277 s), but was always > 120 s. All subsequent analyses were performed in ROIs in the parieto-occipital cortex (10 parietal, 12 occipital and 2 posterior cingulate ROIs) and hippocampus (left and right hippocampal ROIs) of the AAL atlas (Supplementary Table S1). The parieto-occipital ROIs were selected not only because of the important contribution to the dominant alpha rhythm of the brain in the resting-state, but also because of the functional relevance in brain networks and relation with cognitive decline in AD^77–79^. The left and right hippocampi were included too as it is functionally affected early in the course of AD and its dysfunction contributes to cognitive impairment^2,22,44,80^.

After down sampling the fully preprocessed and artefact-free data by a factor 4, the first 10 epochs of 13.12 s each (4096 samples per epoch) of each subject were selected for spectral power analyses. For each epoch and for each ROI in the parieto-occipital cortex and hippocampi, a broad-band power spectrum was computed via a discrete fast Fourier transform (1–48 Hz with steps of 0.076 Hz) using the in-house developed software Brainwave (version 0.9.163.26, available from home.kpn.nl/stam7883/brainwave.html). The power spectra were averaged across epochs and ROIs within the parieto-occipital cortex and the hippocampi to obtain one power spectrum per brain region, per subject. Power spectra noise reduction was performed by constructing the average of 20 consecutive samples of the spectrum, which span a frequency range of ~ 1.5 Hz, for each data point (movmean.m in MATLAB (version R2018b)).

Using the artefact-free but not down sampled MEG data (1250 Hz), we computed the detrended fluctuation analyses (*DFA*) and the functional excitation–inhibition (*fE/I*) over the extended alpha frequency band (6–13 Hz) for each ROI in the parieto-occipital cortex and the hippocampi. Specifically, the alpha band was analyzed because of its relatively high signal-to-noise ratio (SNR), required for reliable *DFA* and thus *fE/I* estimation^35,81^. In addition, the *fE/I* algorithm has been established using simulated alpha oscillations and validated using empirical EEG data filtered in the alpha band. Furthermore, we used the extended version of the alpha frequency band because AD patients typically show progressive oscillatory slowing of the EEG/MEG^41^ and individual alpha peak frequencies have previously been identified in frequencies as low as ~ 6 Hz in AD^82,83^.

Although there are good reasons to analyze the data in the (extended) alpha band (6–13 Hz), estimating the *DFA* and *f/EI* in an a priori selected frequency band might bias the results. Inspired by previous work^40^, we also explored the *DFA* and *fE/I* measures across a broader frequency range (1–44 Hz with steps of 1 Hz) in the parieto-occipital cortex. In theory, one could find a simultaneously in- and decrease or lack of difference in *DFA* and *fE/I* across different frequencies and it remains uninvestigated what this would mean in terms of underlying changes in E–I balance. Therefore, we here present these exploratory results in the supplementary material S1 and limit the main analyses to the alpha band filtered data.

All *DFA* and *fE/I* results were averaged across ROIs within each brain region to obtain one *DFA* and *fE/I* value per brain region per subject. *DFA* and *fE/I* computations were performed in MATLAB (version R2018b) using previously developed scripts^40^ available at https://figshare.com/authors/Simon_J_Houtman/11781767.

### Detrended fluctuation analyses

Long-range temporal correlations are accurately estimated with the *DFA*^84^, robustly observed in occipital alpha oscillations in humans^81,85,86^, and known sensitive to E–I. *DFA* exponents of 0.5 are characteristic of a noisy, uncorrelated, signal, whereas exponents higher or lower than 0.5 indicate positive and negative autocorrelations, respectively. Using a computational network model that generates alpha oscillations, it has been shown that long-range temporal correlations are maximal when there is a balance between excitatory and inhibitory signaling^87^ and that deviations from this balance lead to a decrease in *DFA* exponents. Based on this finding, it is hypothesized that a decrease in *DFA* exponent indicate E–I imbalance, however, it does not provide information about the direction of the change in E–I ratio. For a detailed description of *DFA* computation, we refer to the Supplementary Material S1 as well as^35,85^.

### Functional excitation/inhibition

The *fE/I* algorithm combines signal amplitude envelope and a short time-scale equivalent of the *DFA* exponent to identify functional network E/I ratio. This method was first described by^34^ and we refer to this study and the Supplementary Material S1 for a comprehensive description of the algorithm. Whereas *fE/I* ~ 1 represents neuronal dynamics of a network that is in balance, *fE/I* < 1 indicates an inhibition-dominated regime, and *fE/I* > 1 an excitation-dominated regime. For time series without substantial long-range temporal correlations (i.e. *DFA* < 0.6) amplitude and the fluctuation function do not correlate, and, therefore, the *fE/I* cannot be reliably calculated^34^. To avoid spurious *fE/I* values of ~ 1, a *DFA* threshold was applied which lead to a number of ROIs for which no *fE/I* can be calculated. To find a balance between the number of ROIs that are included and a reliable *fE/I*, we used a *DFA* threshold of 0.55. However, analyses were repeated using the more conservative threshold of 0.6 (Supplementary Fig. S1).

### Aperiodic exponent

We also applied the *FOOOF* algorithm^32^ which computes the aperiodic (1/f-like) exponent of the power spectrum as an index of E–I balance^33^. In short, the *FOOOF* algorithm identifies peaks in the power spectrum and separates the aperiodic component from the periodic oscillations to enable a precise estimation of the spectral exponent. More details about the method are provided in the Supplementary Material S1 and can be found in^32^. Although a substantial number of studies have investigated the aperiodic slope of the power spectrum^32,33,40,62^, there is no agreement on which frequency range of the power spectrum should be used to fit slopes and whether or not to use a ‘knee’ in the fitted slope in order to obtain the best indicators of E–I balance. However, Gao et al.^33^ related an increase in E–I ratio to a flatter slope of the spectrum, in the gamma range specifically, in a computational model and validated this in experimental data from two species. Based on this study, we fit the *FOOOF* to the gamma frequency range (30–48 Hz). The *FOOOF* algorithm is freely available on GitHub (https://github.com/FOOOF-tools/FOOOF*; python 3.7*).

### Statistical analyses

Statistical analyses to compare groups were performed in SPSS (IBSM SPSS Statistics, version 28) or GraphPad Prism (version 9.3.1). The data were inspected for outliers (i.e., those values that are 3 times the interquartile range above the third or below the first quartile) to exclude from final analyses. Subjects with a bad fit for the aperiodic exponent (*R*^*2*^ < 0.8) were excluded from the aperiodic exponent analyses. Assumptions for normality and homogeneity of regression slopes were visually evaluated when appropriate. Demographic group differences were analyzed using chi-square or one-way ANOVA tests. Mean differences in power for each frequency between each group and HE were tested with multiple Mann–Whitney U tests and controlled for the False Discovery Rate (*FDR*) by means of a two-stage step-up method of Benjamini, Krieger and Yekutieli^88^ and using a desired *FDR* of 1.0%. Because after visual inspection of the data at least one clinical group showed non-normal distributions per E–I outcome measure (*DFA*, *fE/I* and aperiodic exponent), overall group differences were tested with nonparametric Kruskal–Wallis tests and pairwise differences between each group and HE were tested with multiple Mann–Whitney U tests. The associations between measures of E–I and MMSE per (elderly) group and in a combined group with all amyloid-positive subjects were investigated by means of Spearman’s rank correlation analyses, and *p* values of 0.05 were considered statistically significant.

## Supplementary Information

Supplementary Information.

## Data Availability

Due to privacy regulations of human subjects, we can only provide the MEG files of the subjects included in our study upon reasonable request and formal data sharing agreement. The *DFA* and *fE/I* algorithms are publicly available at https://github.com/annevannifterick/fEI_in_AD.
