# Supplementary material for: Resting-state oscillations reveal disturbed excitation–inhibition ratio in Alzheimer’s disease patients
Source: Sci Rep. 2023 May 7;13:7419. doi: 10.1038/s41598-023-33973-8 (PMC10164744; doi:10.1038/s41598-023-33973-8)
Supplement: Supplementary file 1 — Supplementary Information. [file 41598_2023_33973_MOESM1_ESM.docx]

Resting-state oscillations reveal disturbed excitation-inhibition ratio in Alzheimer’s disease patients

Anne M. van Nifterick, Danique Mulder, Denise J. Duineveld, Marina Diachenko^3^, Philip Scheltens, Cornelis J. Stam, Ronald E. van Kesteren, Klaus Linkenkaer-Hansen, Arjan Hillebrand, Alida A. Gouw^/^

# Supplementary Methods

***DFA***

In short, the algorithm applies a band-pass filter (here: 6–13 Hz) to a signal by means of a 0.33^th^ order Hamming-window FIR-filter. The amplitude envelope is computed by taking the absolute of the Hilbert transform, the resulting signal is demeaned, and its cumulative sum is computed to create a signal profile. Then, the profile is split into overlapping time windows of lengths that are equally spaced on a logarithmic time-scale, and the linear trend is removed from each window using a least-squares fit. In this study, we applied a range of window lengths between 4.2 and 20 seconds with a 50% overlap between consecutive time windows. The standard deviation (SD) of the detrended signal per window indicates the level of fluctuation around the mean. The average SD was derived across all identically sized windows for each window length. The computed average SDs were then plotted as the function of window length on a log-log scale, and the slope of the trend line was calculated using the least-squares linear fit between window lengths of 4.2 and 20 seconds to obtain the DFA exponent. Smaller time-scales were not fit to avoid a bias of the exponent from temporal correlations induced by the FIR-filter [1].

***Functional Excitation-inhibition algorithm***

As for the *DFA* estimation, the *fE/I* algorithm applies a band-pass filter (between 6 – 13 Hz and a filter order of 0.33 (according to the following: 2 divided by the high-pass filter (6Hz))) to the source-reconstructed data and computes the amplitude envelope. After demeaning the envelope, the cumulative sum of the envelope is calculated. It is then split into time windows with 80% overlap using a window size of 5 times the sample frequency (1250 Hz). Then, the demeaned envelope is normalized by dividing each window by the original amplitude and detrended for each time window. As for the *DFA* analyses, the fluctuations in the demeaned, normalized, and detrended amplitude envelope are captured in the SD. It results in a normalized fluctuation function (*nF*(*t*)) on a short time-scale (*i.e*., 5 s) as an approximation of the *DFA* exponent of the signal. Finally, the *nF*(*t*) is correlated with the amplitude, and the correlation coefficient is subtracted from 1 to obtain the *fE/I* measure.

***FOOOF***

As described in detail by [2] Gaussians were fit to a *log-log* power spectrum to determine the peaks that are due to oscillatory activity. These peaks were subtracted from the spectrum to finally apply an exponential regression line to the remaining aperiodic spectral component. In a subset of data (hippocampal spectra of HY and AD) we explored the number of peaks and the fit (*R^2^*) when allowing 2, 4 or 6 peaks. The fit was similarly good for 4 and 6 peaks. To avoid overfitting peaks but obtain a good fit we set the number of maximum peaks at 4. Peak width limit was set between 2 and 12 units of power and minimum peak height to 0. We did not expect a knee in the gamma frequency range and thus set the aperiodic mode to ‘fixed’.

# Supplementary Results

**
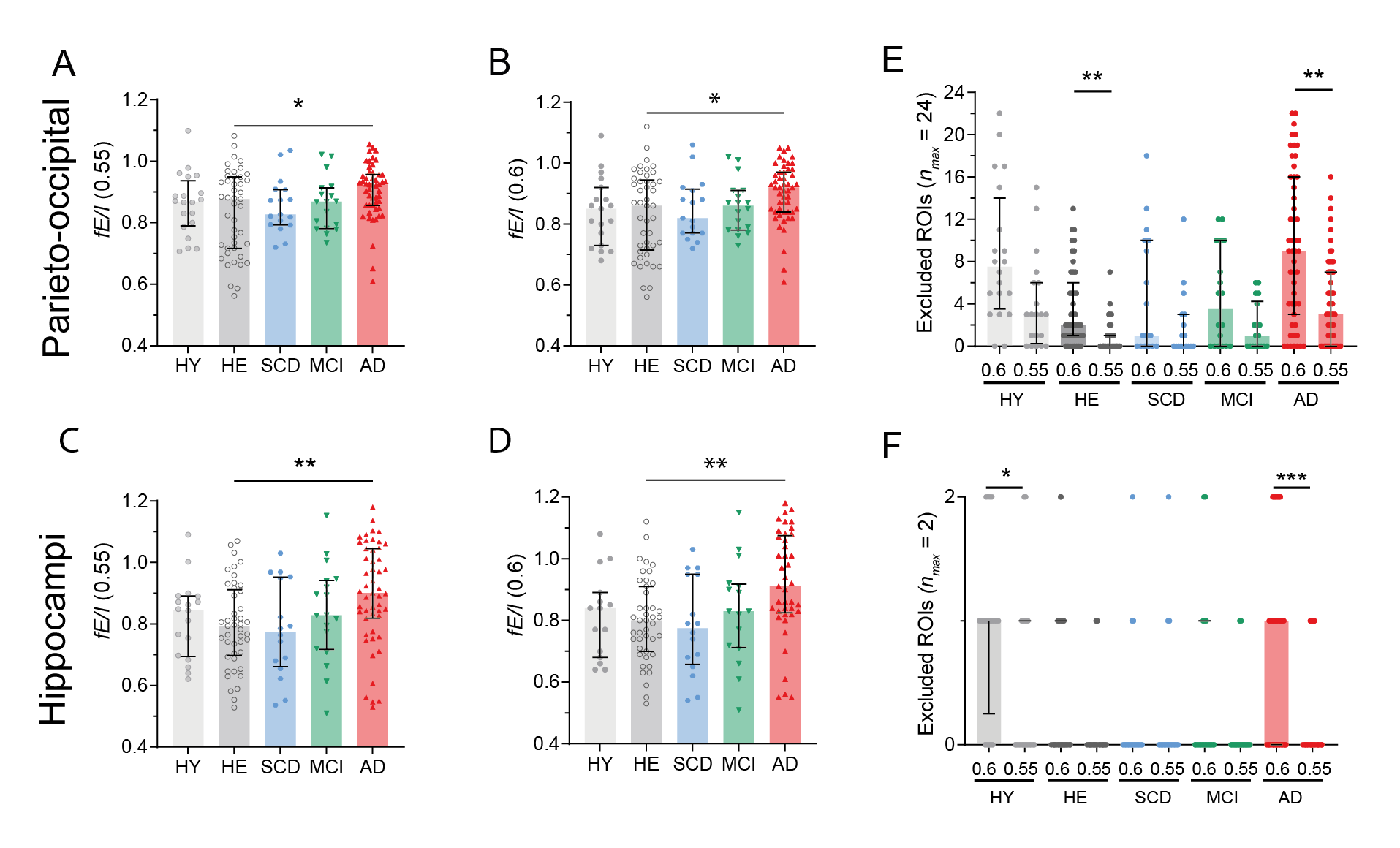
**

**Supplementary Figure 1. *fE/I* across the AD continuum using *DFA* thresholds of 0.55 versus 0.6. (A, B, C, D)** Group differences in *fE/I* using a *DFA* threshold value of 0.55 are comparable to differences found using a threshold of 0.6: *fE/I* is higher in AD patients compared to HE in both parieto-occipital cortex and hippocampi. Also, all groups show median *fE/I* < 1.0 using a threshold of 0.55 as well as 0.6. The *fE/I* of each group (HY, SCD, MCI, AD) was compared to HE using the Kruskal-Wallis test. **(E)** The number of parieto-occipital ROIs for which no *fE/I* values were calculated are significantly lower using a threshold of 0.55 compared to threshold of 0.6 in HE and AD. **(F)** The number of hippocampal ROIs for which no *fE/I* can be calculated is significantly lower using a threshold of 0.55 compared to a threshold of 0.6 in HY and AD. The number of ROIs between a threshold of 0.6 versus 0.55 was statistically compared using multiple Kruskal-Wallis tests. *DFA*, Detrended Fluctuation Analyses; *fE/I*, functional excitation inhibition; NaN, not a number; HY, Healthy Young Controls; HE, Healthy Elderly Controls; SCD, Subjective Cognitive Decline; MCI, Mild Cognitive Impairment; AD, dementia due to Alzheimer’s Disease; *, *p* < .05; **, *p* <.01, ***, *p* <.001.


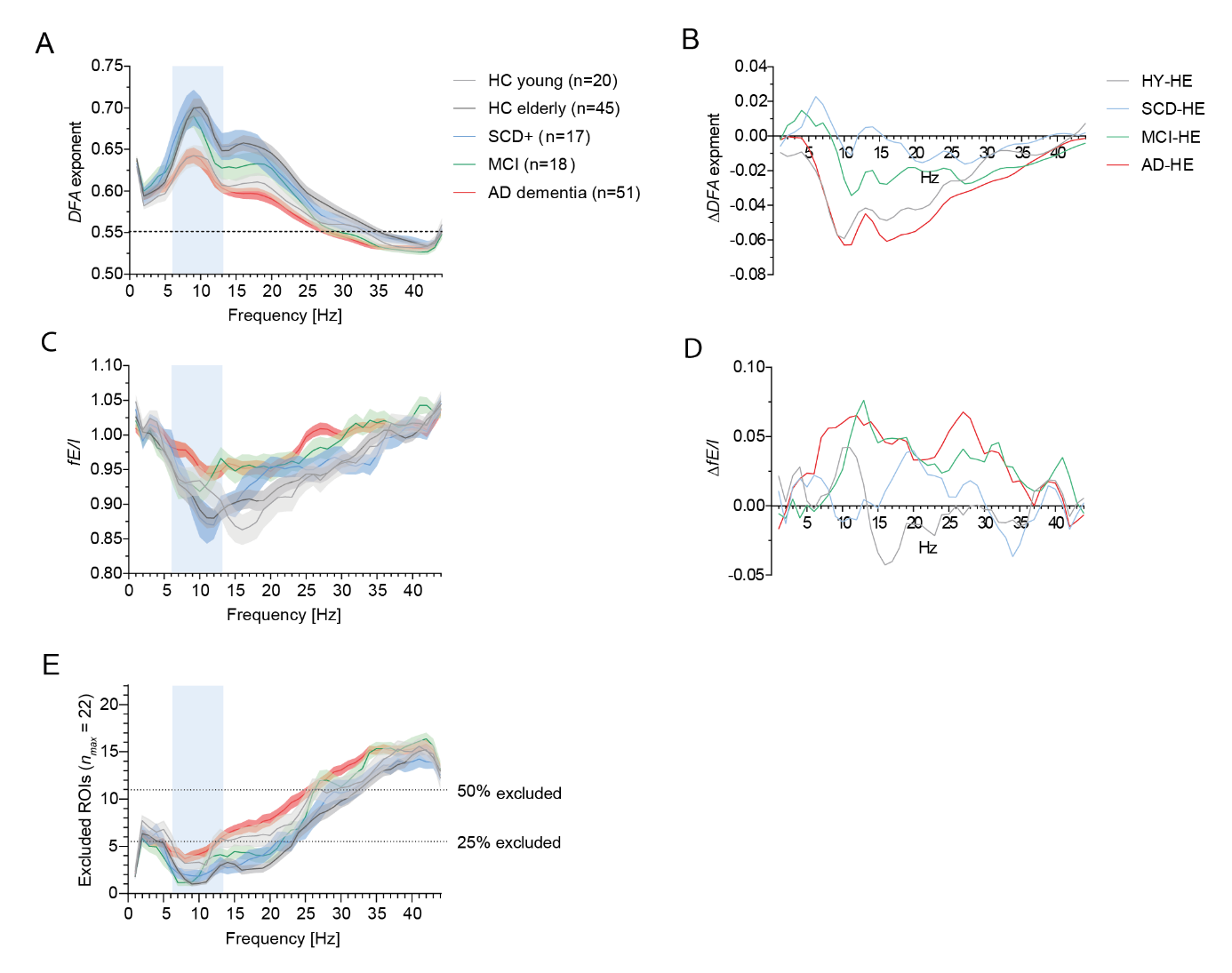


**Supplementary Figure 2. Spectral *DFA* and *fE/I* in patients across the AD continuum.** *DFA* and *fE/I* values were estimated across a broad frequency range (1 – 44 Hz with steps of 1 Hz) in ROIs of the left and right parieto-occipital cortex only and averaged to obtain one value per subject. Shown are the mean and standard error of the mean per group. The blue vertical bars indicate the frequency band (6 – 13 Hz) used in the main analyses. **(A)** *DFA* exponents are highest across the alpha frequencies and show a lower peak in the beta band. **(B)** *DFA* group differences (compared to HE) are greatest across alpha and beta frequencies. **(C)** *fE/*I values are highest in the lowest (~1 – 4 Hz) and highest frequencies (~30 – 44 Hz) analysed, where *DFA* values are lowest. All groups show lowest *fE/I* values across the alpha/beta frequencies (~6 – 30 Hz), where *DFA* values are highest. **(D)** *fE/I* group differences are of similar direction across higher frequencies as found in the broader alpha band used in the main analsyes (*e.g*. AD patients show higher *fE/I* values compared to HE). MCI patients show higher *fE/I* compared to HE in frequencies >13 Hz, which was not captured in the main analyses. **(E)** The average number of ROIs in the parieto-occipital cortex that were excluded from *fE/I* analyses because of low/no long-range temporal correlation (*DFA* < 0.55) is highest across beta - gamma frequencies (20 – 44 Hz) and lowest in the alpha frequencies (~6 – 13 Hz) for all groups. No statistical tests were performed. *DFA*, Detrended Fluctuation Analyses; *fE/I*, functional excitation inhibition; *HY*, Healthy Young Controls; *HE*, Healthy Elderly Controls; *SCD*, Subjective Cognitive Decline; *MCI*, Mild Cognitive Impairment; *AD*, dementia due to Alzheimer’s Disease.

**Supplementary Table 1.** Region number, name and corresponding brain lobe according to the AAL atlas, ordered according to [3].

| **Brain lobe** | **ROI#** | **Region name left hemisphere** | **ROI#** | **Region name right hemisphere** |
| --- | --- | --- | --- | --- |
| Frontal | 1 | Rectus_L | 40 | Rectus_R |
|  | 2 | Olfactory_L | 41 | Olfactory_R |
|  | 3 | Frontal_Sup_Orb_L | 42 | Frontal_Sup_Orb_R |
|  | 4 | Frontal_Med_Orb_L | 43 | Frontal_Med_Orb_R |
|  | 5 | Frontal_Mid_Orb_L | 44 | Frontal_Mid_Orb_R |
|  | 6 | Frontal_Inf_Orb_L | 45 | Frontal_Inf_Orb_R |
|  | 7 | Frontal_Sup_L | 46 | Frontal_Sup_R |
|  | 8 | Frontal_Mid_L | 47 | Frontal_Mid_R |
|  | 9 | Frontal_Inf_Oper_L | 48 | Frontal_Inf_Oper_R |
|  | 10 | Frontal_Inf_Tri_L | 49 | Frontal_Inf_Tri_R |
|  | 11 | Frontal_Sup_Medial_L | 50 | Frontal_Sup_Medial_R |
| Central | 12 | Supp_Motor_Area_L | 51 | Supp_Motor_Area_R |
|  | 13 | Paracentral_Lobule_L | 52 | Paracentral_Lobule_R |
|  | 14 | Precentral_L | 53 | Precentral_R |
|  | 15 | Rolandic_Oper_L | 54 | Rolandic_Oper_R |
|  | 16 | Postcentral_L | 55 | Postcentral_R |
| Parietal | 17 | Parietal_Sup_L | 56 | Parietal_Sup_R |
|  | 18 | Parietal_Inf_L | 57 | Parietal_Inf_R |
|  | 19 | SupraMarginal_L | 58 | SupraMarginal_R |
|  | 20 | Angular_L | 59 | Angular_R |
|  | 21 | Precuneus_L | 60 | Precuneus_R |
| Occipital | 22 | Occipital_Sup_L | 61 | Occipital_Sup_R |
|  | 23 | Occipital_Mid_L | 62 | Occipital_Mid_R |
|  | 24 | Occipital_Inf_L | 63 | Occipital_Inf_R |
|  | 25 | Calcarine_L | 64 | Calcarine_R |
|  | 26 | Cuneus_L | 65 | Cuneus_R |
|  | 27 | Lingual_L | 66 | Lingual_R |
| Temporal | 28 | Fusiform_L | 67 | Fusiform_R |
|  | 29 | Heschl_L | 68 | Heschl_R |
|  | 30 | Temporal_Sup_L | 69 | Temporal_Sup_R |
|  | 31 | Temporal_Mid_L | 70 | Temporal_Mid_R |
|  | 32 | Temporal_Inf_L | 71 | Temporal_Inf_R |
|  | 33 | Temporal_Pole_Sup_L | 72 | Temporal_Pole_Sup_R |
|  | 34 | Temporal_Pole_Mid_L | 73 | Temporal_Pole_Mid_R |
|  | 35 | ParaHippocampal_L | 74 | ParaHippocampal_R |
| Cingulum | 36 | Cingulum_Ant_L | 75 | Cingulum_Ant_R |
|  | 37 | Cingulum_Mid_L | 76 | Cingulum_Mid_R |
|  | 38 | Cingulum_Post_L | 77 | Cingulum_Post_R |
| Insula | 39 | Insula_L | 78 | Insula_R |
| Hippocampus | 79 | Hippocampus_L | 80 | Hippocampus_R |

**References**

1. Hardstone, R., et al., *Detrended fluctuation analysis: a scale-free view on neuronal oscillations.* Front Physiol, 2012. **3**: p. 450.

2. Donoghue, T., et al., *Parameterizing neural power spectra into periodic and aperiodic components.* Nat Neurosci, 2020. **23**(12): p. 1655-1665.

3. Gong, G., et al., *Mapping anatomical connectivity patterns of human cerebral cortex using in vivo diffusion tensor imaging tractography.* Cereb Cortex, 2009. **19**(3): p. 524-36.
